# Combined immunotherapy with “anti-insulin resistance” therapy as a novel therapeutic strategy against neurodegenerative diseases

**DOI:** 10.1038/s41531-016-0001-1

**Published:** 2017-01-23

**Authors:** Yoshiki Takamatsu, Gilbert Ho, Wakako Koike, Shuei Sugama, Takato Takenouchi, Masaaki Waragai, Jianshe Wei, Kazunari Sekiyama, Makoto Hashimoto

**Affiliations:** 1 Tokyo Metropolitan Institute of Medical Sciences, 2-1-6 Kamikitazawa, Setagaya-ku, Tokyo, 156-0057 Japan; 2 The PCND Neuroscience Research Institute, Poway, CA 92064 USA; 3 Department of Physiology, Nippon Medical School, Tokyo, 113-8602 Japan; 4 Institute of Agrobiological Sciences, National Agriculture and Food Research Organization, Tsukuba, Ibaraki 305-8634 Japan; 5 Institute for Brain Sciences Research, School of Life Sciences, Henan University, Kaifeng, 475004 China

## Abstract

Protein aggregation is a pathological hallmark of and may play a central role in the neurotoxicity in age-associated neurodegenerative diseases, such as Alzheimer’s disease and Parkinson’s disease. Accordingly, inhibiting aggregation of amyloidogenic proteins, including amyloid β and α-synuclein, has been a main therapeutic target for these disorders. Among various strategies, amyloid β immunotherapy has been extensively investigated in Alzheimer’s disease, followed by similar studies of α-synuclein in Parkinson’s disease. Notably, a recent study of solanezumab, an amyloid β monoclonal antibody, raises hope for the further therapeutic potential of immunotherapy, not only in Alzheimer’s disease, but also for other neurodegenerative disorders, including Parkinson’s disease. Thus, it is expected that further refinement of immunotherapy against neurodegenerative diseases may lead to increasing efficacy. Meanwhile, type II diabetes mellitus has been associated with an increased risk of neurodegenerative disease, such as Alzheimer’s disease and Parkinson’s disease, and studies have shown that metabolic dysfunction and abnormalities surrounding insulin signaling may underlie disease progression. Naturally, “anti-insulin resistance” therapy has emerged as a novel paradigm in the therapy of neurodegenerative diseases. Indeed, incretin agonists, which stimulate pancreatic insulin secretion, reduce dopaminergic neuronal loss and suppress Parkinson’s disease disease progression in clinical trials. Similar studies are ongoing also in Alzheimer’s disease. This paper focuses on critical issues in “immunotherapy” and “anti-insulin resistance” therapy in relation to therapeutic strategies against neurodegenerative disease, and more importantly, how they might merge mechanistically at the point of suppression of protein aggregation, raising the possibility that combined immunotherapy and “anti-insulin resistance” therapy may be superior to either monotherapy.

## Introduction

The number of patients diagnosed with Alzheimer’s disease (AD), Parkinson’s disease (PD), and other age-associated neurodegenerative diseases, is rapidly increasing worldwide and becoming a common cause of death in aging populations.^1,2^ Consequently, increasing costs for medical treatment and nursing care for these patients has become a serious socioeconomic dilemma. Yet, despite this, there have been no effective treatments established to date, which prevent or arrest the progression of neurodegenerative diseases. With only symptomatic therapies presently available, their effects are modest at best, and they are often associated with side effects. Thus, the development of an effective disease-modifying therapy is among the highest priority in neurodegenerative disease research.

Accordingly, the main objective of this paper is to present perspectives toward a new direction for neurodegenerative disease therapy, and we review the literature in a focused manner to reveal interconnections between immunotherapy and “anti-insulin resistance” therapy relevant to AD, PD, and other age-related neurodegenerative conditions. Over the years, the search for true disease modification for these conditions has been elusive, and it is recognized that early and even prodromal disease treatment/prevention timing will be most effective,^3^ but this must rely on biomarker-based diagnostic confirmation of the presence of expected pathologies for such disorders. One such consideration, immunotherapy against protein aggregation, has been well studied for the last two decades in AD but also in PD, through numerous in vivo and clinical studies. Although it has demonstrated limited clinical efficacy in AD, it still remains the most likely candidate for an initial successful disease modification agent. Another paradigm for such disorders, type 2 diabetes (T2DM) is shown to be an important risk factor for both PD and AD, and methods to favorably manipulate insulin signaling pathways and related molecules has shown tremendous interest and some early clinical success. For instance, “anti-insulin resistance therapy”, including glucagon like peptide-1 (GLP-1) receptor agonists, has been successful in pilot studies of PD, and studies are planned for AD and other neurodegenerative diseases.^4^ Even though both are recognized as important facets in the development of future treatments for neurodegenerative disease, either treatment method alone may not be sufficient to overcome the relevant pathogenic processes. Accordingly, we argue that immunotherapy and “anti-insulin resistance” treatments might target interconnected mechanisms toward suppressing the aggregation of amyloidogenic proteins, and in combination, might greatly enhance therapeutic efficiency.

### Immunotherapy against neurodegenerative disease

Mounting evidence has shown that accumulation of aggregates of amyloidgenic proteins, such as Aβ and tau in AD and α-synuclein (αS) in PD, may be central to the pathogenesis of neurodegenerative diseases.^1,2^ According to the notion that neurotoxicity may be attributed to the aggregates of amyloidogenic proteins, particularly oligomers and protofibrils.^5^ Thus, it is reasonable to predict that suppressing expression of amyloidogenic proteins and/or inhibiting protein aggregation may be effective to delay the progression of neurodegenerative disease. Among various amyloid-targeting strategies, immunotherapy has been extensively investigated in AD, and although several clinical trials of immunotherapy have already been completed (Table 1), including passive and active immunization with Aβ,^6^ the results have so far been unsatisfactory.

**Table 1:** Examples of recent clinical trials of immunotherapy in AD and PD

Target protein	Phase	Clinical trials. gov. identifier	Condition	Sponcer	Mechanism	Schedule
Aβ						
Solanezumab	III	NCT01900665	Mild AD	Eli Lilly and Company	Passive	Jul 2013–Oct 2018
Gantenerumab/Solanezumab	II	NCT01760005	Dominantly inherited AD	Washington University School of Medicine	Passive	Dec 2012–Dec 2019
Gantenerumab	III	NCT02051608	Mild AD	Hoffmann-La Roche	Passive	Mar 2014–Mar 2019
Solanezumab	III	NCT02760602	Prodromal AD	Eli Lilly and Company	Passive	Jun 2016–Apr 2021
Tau						
AADvac1	I	NCT02031198	AD	Axon Neuroscience SE	Active	Jan 2014–Dec 2016
AADvac1	II	NCT02579252	AD	Axon Neuroscience SE	Active	Mar 2016–Feb 2019
C2N-8E12	II	NCT02494024	Progressive supranuclear palsy	C2N diagnostics	Passive	Jul 2015–Aug 2016
αS						
AFFITOPE PD01A	I	NCT01885494	PD	Affiris AG	Active	Jun 2013–Feb 2015
BIIB054	I	NCT02459886	PD	Biogen	Passive	May 2015–Jul 2016
PRX002	I	NCT02157714	PD	Prothena Biosciences Limited	Passive (C-terminal αS)	Jun 2014–Oct 2016
AFFITOPE PD03A	I	NCT02267434	Early PD	Affiris AG	Active	Dec 2014–Aug 2016

Some of the recent clinical trials of immunization for amyloidogenic proteins (Aβ, tau and αS) are selected from “clinicalTrials.gov” (https://clinicaltrials.gov): a service of the U.S. National Institutes of Health

#### Current clinical trials of immunotherapy for AD

**Fig. 1:**
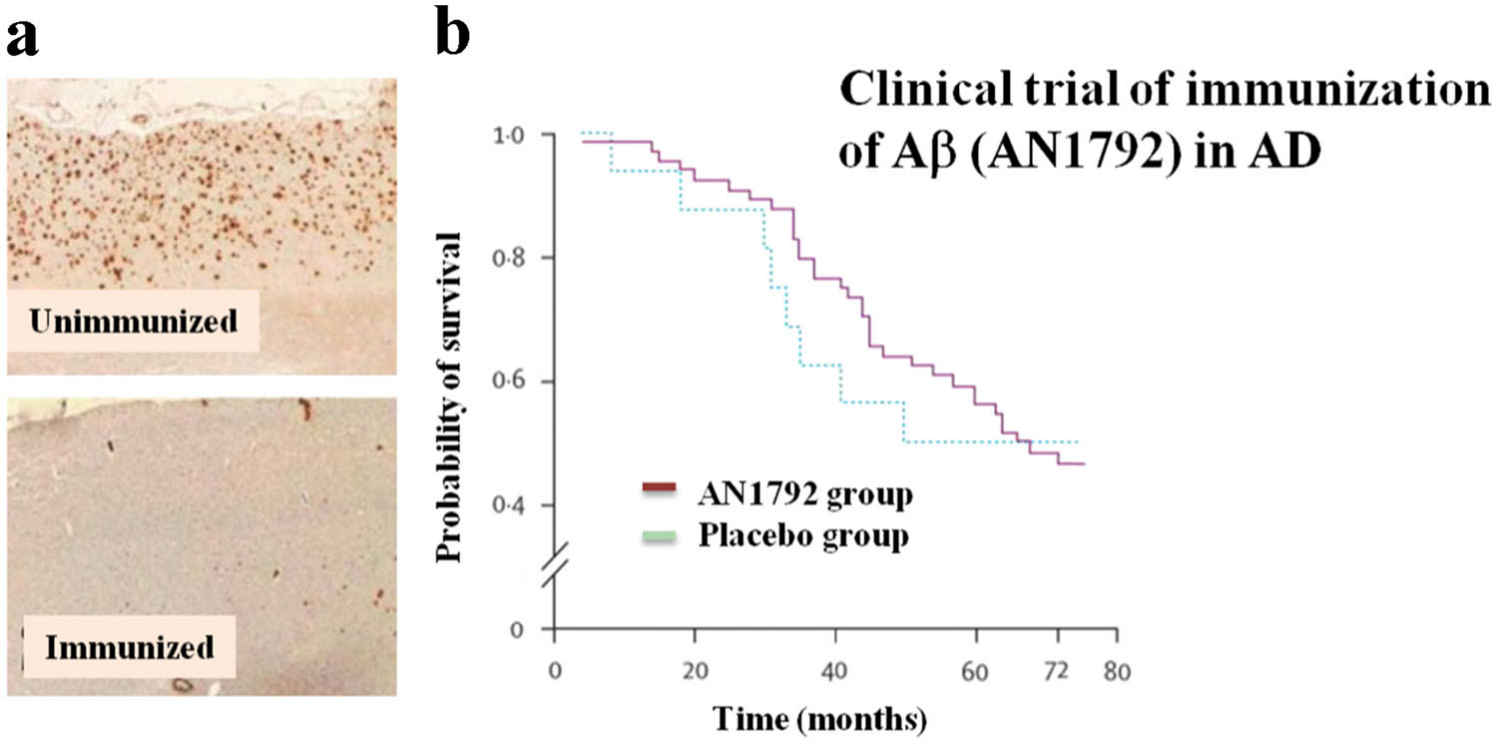
Clinical trial of Aβ immunotherapy for AD. **a** Representative figures of the Aβ immunohistochemistry of the AD autopsy brains with (*low*) or without (*upper*) immunization of Aβ (AN1792). **b** In contrast to histology, there were no significant differences in cognitive functions between AN1792-treated- and placebo treated-groups. Reprinted from Holmes et al. (2008)^7^

Contrary to the positive findings at the preclinical level, results of active amyloid immunotherapy in AD clinical trials have been disappointing. Notably, brains of study participants at autopsy revealed that formation of senile plaques was effectively suppressed by Aβ immunization in AD patients (Fig. 1a), while cognitive performance was not significantly improved (Fig. 1b).^7,8^ Adversely, serious complications, such as meningoencephalitis, were unexpectedly encountered leading to early trial discontinuation.^9^ Presently, the exact reasons behind the lack of a more robust treatment effect remain unclear, and although Aβ immunization proved successful, reduction of amyloid plaque burden failed to lead to substantive improvement in dementia symptoms in AD. Indeed, a discrepancy between amyloid pathology and cognition is an ongoing empirical issue in various aspects of neurodegeneration.^10^ Perhaps one explanation is that Aβ immunotherapy must be initiated at very early stages in the pathogenesis to be of benefit. To examine this hypothesis, new studies, such as “Dominantly Inherited Alzheimer Network”, have begun to evaluate the therapeutic effect of immunization administered at the pre-symptomatic stage of familial AD.^11^ Findings from these studies will be invaluable for AD and for other neurodegenerative diseases, emphasizing that early treatment may be a necessary requirement for disease-modifying therapies.

#### Passive Aβ immunotherapy in clinical trials

Recently, great interest has focused on two passive AD immunotherapy protocols, one using solanezumab, a humanized monoclonal antibody from Eli Lilly and Company against the mid-portion of Aβ recognizing only soluble monomeric amyloid, and another, gantenerumab from Hoffman-La Roche which recognizes only fibrillar Aβ (Table 1). The rationale of each differing greatly, solanezumab seeks to target the soluble Aβ pool as a “sink”,^12^ whereas gantenerumab might stimulate microglial and phagocytic mechanisms to clear larger amyloid species from brain.^13^ Although two independent phase III clinical trials of solanezumab failed to meet their endpoints, pooled data from these two studies demonstrated a statistically significant slowing of cognitive decline in mild AD but not moderate AD.^14^ A confirmation trial of solanezumab is currently underway to determine if the effects are reproducible (Table 1). Similarly, results of a gantenerumab phase II trial also failed to meet primary and secondary endpoints, but a trend toward benefit was noted in posthoc analysis,^15^ and a phase III trial of gantenerumab has been ongoing since 2014 (Table 1). At the present stage of evolution in amyloid immunotherapy, it remains unclear how clinically effective either rationale will be, but ongoing refinements and improvements in efficacy might be expected.

#### Immunotherapy against other amyloidogenic proteins

**Fig. 2:**
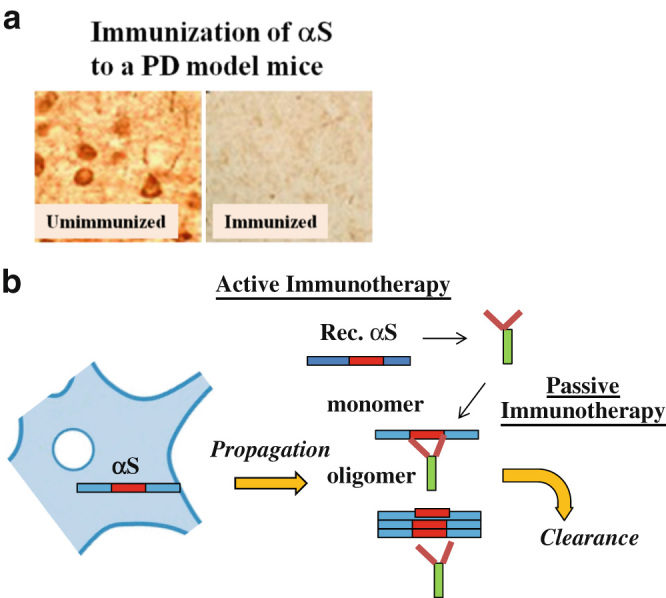
Preclinical results of αS immunotherapy for PD. **a** Representative figures of the αS immunohistochemistry of the transgeic mice brains with (*right*) or without (*left*) immunization of αS. Reprinted from Masliah et al. (2005).^20^ **b** Schematic representation of the mechanism of active and passive immunotherapies of αS

Tau has been well characterized as a major constituent of neurofibrillary tangles (NFT) in AD brain. Since both the number of NFT as well as synaptic density measurements correlate well with memory dysfunction in AD,^16^ it is reasonable to speculate that tau might be a promising therapeutic target for AD and other tauopathies, such as frontotemporal dementia with Parkinsonism linked to chromosome-17, cortico-basal degeneration, Pick’s disease and progressive supranuclear palsy.^17^ Indeed, immunotherapy targeting pathological tau prevented cognitive decline in P301 tangle model mice,^18^ and based on these promising findings, clinical trials of tau immunotherapy are ongoing (Table 1).^19^ Furthermore, in PD, Masliah et al. showed that active immunization with recombinant αS was effective in reducing neuronal accumulation of αS aggregates in mice expressing human wild type αS (Fig. 2a).^20^ More recently, they showed that passive immunization with a monoclonal antibody against the C-terminus of αS was effective at reducing the behavioral deficits in a water maze and significantly reduced histopathology in mice expressing the C-terminus of human wild type αS.^21^ This has prompted ongoing early phase clinical trials of passive αS immunotherapy to explore safety, pharmacokinetics and efficacy in PD (Table 1).^22^ It is predicted that tau and αS may be mutually associated in neurodegeneration. Supporting this notion, a genome wide association study revealed that the tau gene is associated with PD in Asian populations.^23^ Moreover, it was previously demonstrated that αS synergistically promotes fibrillization of tau in vitro and in mice models.^24^

Because both tau and αS are cytosolic proteins, it must still be determined how intracellular amyloidogenic proteins become immunized. With regard to this, one possible mechanism is that the antibody, either active or passive, might recognize αS on the cell membrane and that the antibody-αS complex is endocytosed and targeted to the lysosomal pathway,^20^ whereby antibody recognition of αS may be dependent on co-expression of a major histocompatibility complex class I (MHC-1) on the neuronal membrane.^25^ Dysregulated MHC-1-restricted, antigen-specific cytotoxic T-cell response might be important to immunotherapy efficacy or its associated adverse effects. Thus, further studies are necessary to elucidate the underlying mechanism of immunization in this context. Alternative, but not a mutually exclusive mechanism is that the antibody may target an extracellular αS pool, since αS may transfer from one neuronal population to another via a prion-like mode of transmission (Fig. 2b).^26^ It is conceivable, therefore, that a similar mechanism may also apply to tau immunotherapy. Finally, in addition to Aβ, tau and αS, immunotherapies are being considered for other amyloidogenic proteins, such as SOD1, TDP-43, huntingtin and PrP proteins.^27–29^

### Enhancing immunotherapy for neurodegenerative disease

The mechanisms underlying immunotherapy for neurodegenerative disorders, such as PD and AD, are revealed to be more complex than previously imagined, and its effectiveness may depend upon subtle considerations that perhaps have not been discussed previously. We content that in order to increase the effectiveness of immunotherapy, perhaps several key factors related to the nature of the immunization target must be considered.

#### Toxicity of oligomers and protofibrils

Recent studies suggest that protein aggregates are heterogeneous in composition. Some aggregates, such as oligomers and protofibrils, are highly toxic, while other species, including mature fibrils, lack toxicity.^5^ Assuming that both oligomers and protofibrils are precursors of mature fibrils,^5^ it is possible that anti-aggregation treatments may shift the equilibrium of protein aggregation from mature species to smaller toxic species, such as oligomers and protofibrils. As a result, suppression of protein aggregation might adversely enhance neurotoxicity, exacerbating synaptic dysfunction by disrupting LTP, axonal transport, and synaptic vesicle activity, eventually leading to cognitive, behavioral and motor deficits.^10,30^ Given such a paradoxical effect of immunotherapy, it is proposed that the toxic species, oligomers and protofibrils, rather than other forms, should be specifically targeted. Since oligomers and protofibrils have been examined most extensively using in vitro studies, further in vivo confirmation of this hypothesis is required. Similarly, the more toxic species of αS might be regarded as another specific target of immunotherapy.^31^

#### Other amyloidogenic proteins influencing immunotherapy

Moreover, the interaction among the various amyloidogenic proteins might be another important influence in the neurodegenerative process, which affects immunotherapy efficiency. For instance, αS aggregation can be induced by Aβ,^32^ and αS was also shown to stimulate the aggregation of PrP protein, increasing prion infectivity.^33^ Therefore, aggregation and interaction of amyloidogenic proteins may result in protein cross-seeding, accelerating disease progression. Alternatively, interfering with any single or multiple amyloidogenic proteins may indirectly affect the structure and function of other co-aggregating proteins, causing enhanced oxidative stress and dysregulation of signal transduction. With this in mind, simultaneous targeting of multiple amyloidogenic proteins may be even more therapeutically effective when compared with single protein immunization. Therefore, one unique method may be to utilize a “mixed immunotherapy” strategy comprised of multiple antibodies corresponding against different amyloidogenic proteins. Another alternative immunotherapy might utilize an antibody, which broadly recognizes β-sheet structures, a common feature of many amyloidogenic protein assemblies.

#### β-Synuclein and the regulation of protein aggregation

β-synuclein (βS), the non-amyloidogenic homolog of αS, is abundantly expressed in the brain, co-localized with αS in the presynaptic terminal.^34^ Although wild type βS negatively regulates αS aggregation,^34^ βS containing in dementia with Lewy bodies (DLB)-linked familial mutations P123H and V70M are prone to aggregate and co-promote the aggregation of αS,^35,36^ leading to neurotoxicity. This raises the possibility that wild-type βS might also be converted from an inhibitor of aggregation to an aggregation-promoting protein through other structural alterations due to pathological aging or exposure to environmental factors which ultimately contributes to αS pathology.

Given the intriguing reciprocal regulatory relationship between αS and βS, it stands to reason that perhaps other examples exist in biology of similar amyloidogenic and non-amyloidogenic protein pairings. In fact, such relationships can be observed vertically in all amyloidogenic proteins and their homologous partners with functional and pathological implications.^37^ If non-amyloidogenic proteins are critical negative regulators of aggregation of their amyloidogenic protein partners, then structurally altered non-amyloidogenic proteins might serve as attractive targets for immunotherapy. For instance, with potential relevance for AD, amyloid-like precursor protein 2 was also shown to inhibit the oligomerization of amyloid precursor protein (APP), leading to suppression of Aβ protein expression.^38^

#### Physiological function of amyloidogenic proteins

Furthermore, and perhaps the most important factor for immunotherapy of PD and DLB, the normal physiological function of αS must be considered. Since it is predicted that aggregation of amyloidogenic proteins is accompanied by both “gain of toxicity” and “loss of function” of neurotrophic/neuroprotective properties, elimination of αS neurotoxicity may be insufficient for effective immunotherapy, but also requires the restoration of neuroprotective mechanisms. To address this, the normal physiologic function of αS must be better understood. Because αS was previously identified as a molecule critical for the learning of songs in canaries, αS may be an important molecular mediator of learning and memory.^39^ Consistent with this view, it was shown that αS regulates the size of the presynaptic vesicular pool in primary hippocampal neurons.^40^ Moreover, αS promotes SNARE-complex assembly in vivo and in vitro neurons.^41^ However, αS knockout mice revealed no specific phenotype,^42^ with large accumulations of αS present in hematopoietic cells, such as red blood cells,^43^ suggesting that αS might have as yet undefined biological functions.

Similarly, in AD, the functions of APP and Aβ remain poorly understood. APP is a large transmembrane protein that is ubiquitously expressed in many tissues, including brain.^1^ Although the physiological functions of APP remain unclear, accumulating evidence suggests that secrteted APP alpha (sAPPα) may be involved in a number of biological processes, including cell proliferation, adhesion, neurite extension and cellular trafficking.^44^ Notwithstanding the importance of the “loss of function” of sAPPα in AD, the current immunotherapy for AD focuses only on reducing levels of brain Aβ, and although Aβ and sAPPα are both normal, soluble products of cellular metabolism,^45^ the physiological functions of Aβ remain unclear.

#### Co-factors affecting protein aggregation

Next, co-factors have also been identified which directly or indirectly stimulate the aggregation of amyloidogenic proteins, such as Aβ and αS. For example, it is well known that some minerals, such as zinc, iron and aluminum, interact directly with amyloidogenic proteins to induce aggregation.^46,47^ Moreover, multiple other risk factors for familial neurodegenerative diseases also indirectly stimulate amyloidogenic protein aggregation. Thus, the elimination of mineral elements and other secondary factors may lead to further enhancement of immunotherapy protocols.

### Diabetes and other metabolic disorders increase risk of neurodegenerative diseases

**Fig. 3:**
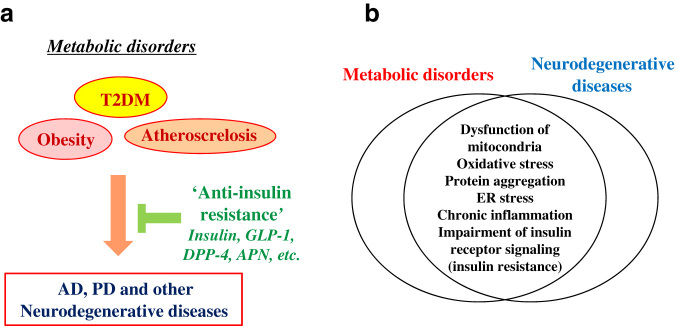
Metabolic dysfunctions exacerbate neurodegenerative diseases. **a** A schematic figure showing that neurodegenerative diseases, including AD and PD, are stimulated by metabolic disorders, such as obesity, T2DM and atherosclerosis. Accordingly, “anti-insulin resistance” therapies, including insulin, GLP-1, dipeptidyl peptidase-4 (DPP-4)﻿ and adiponectin (APN), are therapeutic strategies against neurodegenerative diseases. **b** A schematic figure showing that some pathogenic mechanisms are overlapped between metabolic disorders and neurodegenerative diseases. These include mitocondrial dysfunction, oxidative stress, protein aggregation, ER stress, chronic inflammation, impairment of insulin receptor signaling (insulin resistance), etc.

T2DM and other metabolic disorders, such as obesity and atherosclerosis, are well-recognized epidemiological and clinical risk factors for AD (Fig. 3a).^48^ The risk of T2DM for other neurodegenerative conditions, including PD, has been inconsistently reported. Although some reports described increased risk of T2DM in PD,^49,50^ a recent meta analysis have shown that diabetic individuals have a decreased incidence of PD despite significant heterogeneity.^51^ Furthermore, it is increasingly recognized that T2DM promotes a wide range of brain disorders, including neuropsychiatric disease and ischemia.^52^ Thus, it is likely that T2DM may be a common pathogenic contributor which exacerbates many disorders of the central nervous system.^53^

Evidence from animal studies supporting a role for T2DM in neurodegeneration also parallel clinical and epidemiological observations. For instance, cross-bleeding of mice expressing Swedish APP with diabetic mice (either ob/ob or NSY mice) revealed that a variety of neuropathology, including cerebrovascular inflammation and severe amyloid angiopathy, was exacerbated, while the diabetic phenotypes in cross-bred mice are accelerated compared with those of monogenic mice, suggesting that AD and diabetes induce mutual disease progression.^54^ Relevant to PD, diet-induced obesity accelerates the onset of “terminal phenotypes” in αS transgenic mice characterized by increased amyloid-like deposits and premature αS pathology.^55^

The mechanisms by which T2DM exacerbate neurodegenerative diseases are still elusive. In this regard, there are at least several possibilities that are not mutually exclusive. First, neurodegenerative disorders and T2DM share similar pathologies, including protein aggregation, oxidative stress due to mitochondrial dysfunction, ER stress and inflammation (Fig. 3b).^56,57^ In addition, it was shown that expression of the diabetes-related genes is altered in AD brains,^58^ suggesting that AD and T2DM might share related mechanisms. Second, a number of studies have shown that diabetes-related vasculopathies, particularly atherosclerosis, may underlie the progression of neurodgeneration and clinical dementia.^59^ Indeed, vascular Aβ deposition manifesting as congophilic angiopathy is a consistent pathological feature in AD brain.^60^ Third, it has been assumed that formation of advanced glycation end products (AGEs) may commonly underlie among T2DM and other age-related disorders, including neurodegenerative diseases.^61^ In support of this notion, AGEs modification was characterized in various lesions, such as senile plaques, NFTs, and cerebral amyloid angiopathy in AD^62^ and Lewy bodies in PD.^63^ Consistent with this, it was shown that AGEs modification adversely affect folding, oligomeric, and DNA binding properties in amyloidogenic protein.^64^

**Fig. 4:**
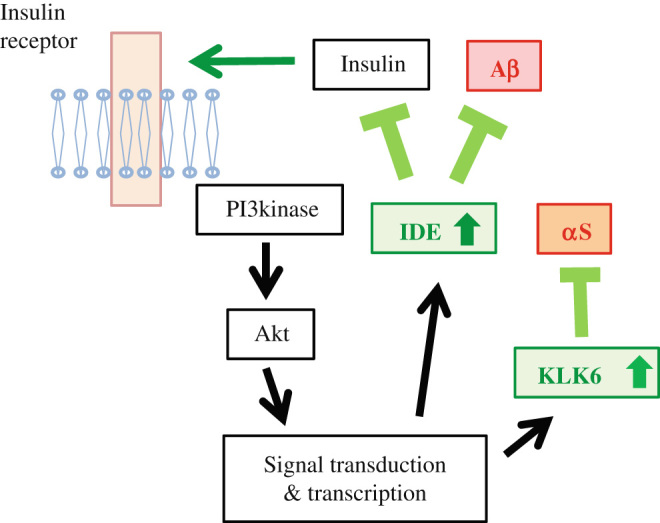
Insulin receptor signaling activates proteases for amyloidogenic proteins. A schematic figure showing that insulin receptor signaling pathway targets IDE and degrades both insulin and Aβ. Similarly, KLK6 may be regulated by insulin receptor signaling pathway to degrade αS

Fourth, and perhaps most importantly, insulin signaling may be impaired in both T2DM and neurodegenerative disorders. At the molecular level, in, insulin receptor signaling may be impaired primarily at the level of insulin receptor substrate (IRS)-1/2.^65^ Given that efficient insulin signaling is essential for neuronal survival, the loss of this critical pathway may therefore lead to neurodegeneration. Furthermore, the loss of function of insulin receptor signaling may lead to stimulate protein aggregation. It has been characterized that insulin degrading enzyme (IDE), which degrades both insulin and Aβ, is up-regulated by insulin receptor signaling,^66,67^ but degradation of insulin subsequently down-regulates insulin receptor signaling, serving as a negative feedback mechanism on IDE.^67^ Thus, it is predicted that dysregulated activity of the insulin receptor signaling pathway may lead to increased Aβ accumulation (Fig. 4). Similar is the case for PD. It was shown that Kallikrein 6 (KLK6), also called neurosin, degrades αS in both cell-based and mouse studies.^68,69^ Although the relationship of insulin receptor signaling with KLK6 is still unclear, it is worth noting that expression and secretion of KLK6 relies on caveolin-1-stimulated Akt, a major signaling molecule in the insulin receptor signaling pathway, in colon cancer cells.^70^ Indeed, it was also shown that caveolin-1 expression was up-regulated in a cellular model of α-synucleinopathies^71^ and in AD brain.^72^ Although confirmation of a similar pathway in brain is still lacking, it is possible that stimulation of the insulin receptor signaling via “anti-diabetes” therapy, may result in KLK6 activation, ultimately leading to αS degradation (Fig. 4). Thus, the mechanisms by which “anti-insulin resistance” therapy suppresses protein aggregation in PD may be partly analogous to that in AD.

### “Anti-insulin resistance” as a therapeutic strategy

Since insulin resistance is central to T2DM pathogenesis, amelioration of abnormal insulin resistance might not only improve T2DM, but also delay the progression of neurodegenerative diseases, including AD and PD. Indeed, evidence suggests that augmenting insulin receptor signaling by direct stimulation of the insulin receptor pathway by insulin and also indirect positive modulation of the pathway by other factors, including incretins and APN, may be effective against neurodegenerative disease (Fig. 3a).

#### Insulin

Increasing evidence indicates that insulin carries out multiple functions in the brain and dysregulation of insulin signaling may contribute to AD and PD.^65^ Insulin exerts a wide variety of biological effects through its interaction with the insulin receptor, a transmembranous glycoprotein receptor tyrosine kinase. Activated insulin receptor phosphorylates the intracellular substrate, IRS-1/2, which associate downstream with p85 subunit, growth factor receptor binding protein 2, and the Syp protein tyrosine phosphatase leading to activate the PI3kinase—Akt pathway to affect many downstream cellular functions.^73^ Importantly, it has been shown that compromised inactivation of GSK-3β by Akt may be involved in tau hyperphosphoryation.^74^ Furthermore, mammalian target of rapamycin (mTOR), which is situated downstream of Akt in insulin signaling, may be an important molecular intersection of aging, diabetes and neurodegenerative diseases.^75^ It has been shown that the phenomena of lifespan extension due to caloric restriction (CR) may be attributed to suppression of mTOR, a cellular sensor of the nutrient environment of the organism.^76^ Although the relevance to human lifespan remains unclear, the effect of CR/starvation on aging has been observed in a range of species from yeast to mammals.^76^ Since ample evidence demonstrates abnormally up-regulated mTOR in AD, it is of great interest to determine whether therapeutically improving insulin resistance may ultimately affect longevity in AD and PD patients.^77^

Reinforcing the concept that overcoming aberrant insulin signaling can be clinically effective in treating neurodegenerative disease (Fig. 3a), Craft et al. found that intranasal delivery of insulin improved working memory and cognitive performance and also cerebral glucose metabolism in mild cognitive impairment and AD.^78^ AD patients with ApoE4/4 genotype particularly benefitted from intranasal insulin, perhaps suggesting an interaction of insulin signaling with the ApoE4 risk factor.^79^ Presently, further clinical trials of intranasal insulin in AD are ongoing (Table 2), and insights gained from these trials will greatly advance “anti-insulin resistance” AD therapy,^80^ providing insight into the pathogenesis through examination of relevant biomarkers in cerebrospinal fluid and brain imaging. It is thus expected that clinical studies using intranasal insulin for PD (Table 2) and other neurodegenerative disorders might also be pursued in future.

**Table 2:** Examples of recent clinical trials of the “anti-insulin resistance” therapy in AD and PD

Agent	Phase	Clinical trials gov. identifier	Condition	Sponcer	Schedule
Intransal insulin	II	NCT01547169	Mild Cognitive Impairment, AD	University of Washington	Mar 2011–Dec 2012
Intransal insulin	II	NCT01767909	Mild Cognitive Impairment	University of Southern California	Sep 2013–Feb 2017
Intransal insulin	II	NCT02064166	PD, Multiple system atrophy	University of Massachusetts, Worcester	Feb 2014–Sep 2015
Intransal insulin	II	NCT02503501	Mild Cognitive Impairment	Health Partners Institute	Aug 2015–Sep 2017
Exenatide	II	NCT01255163	Mild Cognitive Impairment, AD	National Institute on Aging	Nov 2010–Dec 2018
Exenatide	II	NCT01971242	PD	University College, London	Jun 2014–Jun 2016
Liraglutide	II	NCT01469351	AD	University of Aarhus	Jan 2012–Apr 2013
Liraglutide	II	NCT01843075	AD	Imperial College London	Jan 2014–Jan 2017

Some of the recent clinical trials of the “anti-insulin resistance” therapies, such as insulin and GLP-1, in AD and PD, are selected from Clinical trials of GLP-1 in AD and PD “clinicalTrials.gov” (https://clinicaltrials.gov): a service of the U.S. National Institutes of Health

#### Incretins

An important emerging area of interest, bi-directional gut-brain interactions are important to functional gastrointestinal disorders as well as in various CNS diseases, including depression and neurodegenerative conditions.^81^ Recently, studies have highlighted the potential role of GLP-1 in suppressing the pathogenesis of neurodegeneration. GLP-1, a member of the incretin family, is secreted by L cells in small intestinal mucosa in response to food intake and stimulates insulin secretion by pancreatic β-cells.^4^ The cognate receptor of GLP-1 belongs to the family of guanine nucleotide-binding protein-coupled receptors, which are involved in the regulation of many factors including hormones, neurotransmitters and nutrients, such that they are considered attractive drug targets (Fig. 3a).^82^ GLP-1 receptors are widely expressed in neurons and glia throughout the CNS,^83^ suggesting that GLP-1 may both cross the blood-brain-barrier originating from peripheral sources or possibly be synthesized locally in the brain. Because GLP-1 improves insulin resistance without causing hypoglycemia, GLP-1, unlike insulin, might exert neuroprotective effects while safely being administered by many routes.^4^

Indeed, GLP-1 analogs, such as exenatide and liraglutide, already safely in clinical use to treat T2DM, have been extensively studied in clinical trials of neurodegenerative diseases. Notably, exenatide was shown to be effective in a pilot study in PD (Table 2).^84^ In this study, 45 patients with moderate PD were subjected to receive subcutaneous injection of either exenatide or controls for 12 months. As a result, exenatide was well tolerated, clinically relevant improvements in PD across motor and cognitive measures compared with the control group was suggested.^84^ Subsequently, a few separate clinical trials are currently evaluating the safety and efficacy of exenatide and liraglutide in AD (Table 2). One study shows that Aβ load or cognition measures were weak although glucose metabolism was improved,^85^ while the others are expected to yield results in the near future (Table 2).

Finally, inhibiting DPP-4, the primary inhibitory regulator of GLP-1, may be neuroprotective through activation of AMP-activated protein kinase (AMPK) in double transgenic AD mice expressing a chimeric mouse/human APP and a mutant human presenilin 1.^86^ Like GLP-1, DPP-4 is already approved for clinical treatment of T2DM and may be another promising “anti-insulin resistance” treatment for PD and AD (Fig. 3a).

#### APN

APN is a multifunctional adipocytokine that is suppressive on inflammation and is essential to sensitize insulin receptor signaling. It has been shown that the level of APN, is decreased in metabolic syndromes such as, obesity, cardiovascular disease and T2DM.^87^ At the level of animal experimental models, APN is protective against these diseases in addition to other T2DM-related diseases, including atherosclerosis, osteoporosis, and chronic pulmonary obstructive disease.^57,87^ In the nervous system, APN may be neuroprotective against ischemia, depression and other brain conditions.^57,87^

These protective effects of APN are exerted through binding of APN to its receptors, AdipoR1 and AdipoR2,^88^ in which a signaling cascade involving AMPK and peroxisome proliferator-activated receptor-α is activated.^89^ Since APN receptors are abundant in the nervous system,^88^ activation of APN signaling may also be beneficial for treating neurodegenerative diseases (Fig. 3a). To examine this, we recently demonstrated that intranasal administration of APN ameliorated neuropathological features, such as protein aggregation and impaired motor activity, in a transgenic mouse model of α-synucleinopathies.^57^ Subsequently, osmotin, the plant homolog of APN, was also shown to attenuate Aβ42-induced neurotoxicity and tau hyperphosphorylation in hippocampus of wild mice.^90^

It has been shown that exercise ameliorates insulin resistance in various tissues, including neurons.^91^ In the similar context, exercise has been shown to be beneficial for learning and memory in mice^92^ as well as for neurological health in humans.^93^ Given that APN is an exercise mimetic in terms of signal transduction and transcription,^89^ this molecule may have tremendous potential in the therapy of neurodegenerative disorders. One may naturally predict that the therapeutic activation of the APN signaling pathway by pharmacological means may be ideal for bed-ridden elderly to prevent the pathological consequences of extreme inactivity from T2DM, vascular and other metabolic diseases. In this regard, AdipoRon, an AdipoR agonist, was recently isolated as a small molecule which ameliorates the diabetic phenotype in the ob/ob mice,^94^ raising the possibility that AdipoR agonists may be promising therapies for T2DM. Of great interest, it will be important to determine whether the AdipoR agonists are clinically effective for protection against neurodegenerative diseases as well.

### Combined therapy of immunotherapy and “anti-insulin resistance”?

Because disease-modifying monotherapy for neurodegenerative disorders such as AD and PD have not proven to be clinically effective, use of combination therapies has naturally gained attraction. In this context, it was shown that combined anti-Aβ and Liver X-receptor (LXR) antagonist treatment improved cognitive deficits in the mice expressing Swedish type of APP.^95^ Since LXR-α and LXR-β are nuclear hormone receptors that bind oxidized forms of cholesterol, these molecules might play a protective role in AD.^96^ In PD, it was described that a combination of immunotherapy with anti-inflammatory treatment might be a future strategy for the treatment of α-synucleinopathies.^96^ Provided that chronic inflammation promotes both T2DM and neurodegenerative disease, use of anti-inflammatory treatment in neurodegeneration would complement the “anti-insulin resistance” strategy.

**Fig. 5:**
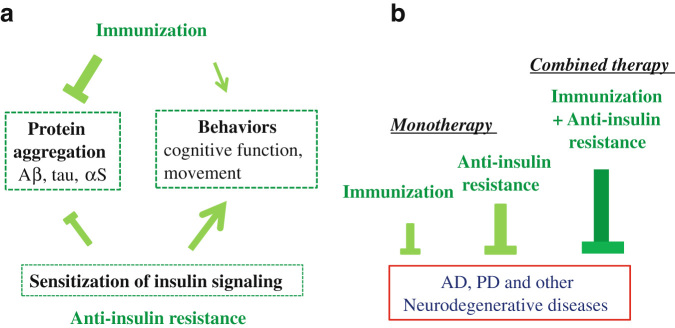
Schematics of the combined immunotherapy with “anti-insulin resistance”. **a** A schematic figure showing that immunization is efficient to suppress aggregation of amyloidogenic proteins, but not to improve behaviors, including cognitive function and movement. On the other hand, “anti-insulin resistance” therapy is beneficial for both protein aggregation and behaviors. **b** Combined immunotherapy with “anti-insulin resistance” therapy may be efficient compared to each monotherapy

In this paper, we contend that anti-aggregation immunotherapy combined with “anti-insulin resistance” treatments might be more efficacious than monotherapies in neurodegenerative diseases (Fig. 5), and the rationale behind this is two-fold. First, it has been characterized that intracellular signaling may be altered during the neurodegeneration. In α-Synucleinopathies, the activities of signaling molecules, such as MAP kinases, might be altered either by direct association of αS^97^ or indirectly via certain signaling modulators.^98^ In this context, reduced expression of amyloidogenic proteins by immunization may lead to the increased efficacy of insulin receptor signaling pathway. Once improved, insulin receptor signaling pathway by itself may suppress protein aggregation through activation of proteases, including IDE and KLK6. Therefore, it is predicted that suppression of protein aggregation by immunization may improve the “anti-insulin resistance therapy”.

Second, empirical evidence indicates that in various aspects of neurodegeneration, histopathological findings, such as protein aggregation and inflammation, are frequently dissociated from cognitive and behavioral performance.^10^ Therefore, any therapeutic strategies against neurodegenerative diseases must have the obligate potential to not only ameliorate neuropathological defects, but also improve or stabilize cognitive and behavioral outcomes. In this regard, accumulating evidence suggests that activation of insulin signaling pathway may ameliorate behaviors (Fig. 5a). In support of this notion, nasal injection of insulin was effective to improve cognitive functions in a pilot study in AD.^78^ Furthermore, GLP-1 analog exenatide was effective in a pilot study in PD.^84^ Thus, given that immunotherapy alone is not efficient for behaviors, sensitization of insulin receptor signaling may be important. Collectively, immunotherapy and “anti-insulin resistance” therapy may be mutually supplemental. One may expect an additive or even synergistic efficiency for the combination of these two therapies (Fig. 5b).

In addition, it is important to consider that a disease modifying therapies may affect the symptom of the disease. In particular, given that Akt is a critical modulator of dopamine receptor signaling in striatal neurons,^99^ chronic stimulation of Akt by the “anti-insulin resistance” therapy, might result in hyper-activation of dopamine signaling in striatum, leading to altered behaviors, such as a deregulation of circadian rhythmus and psychiatric problems. This possibility may be further augmented by the combined therapy with immunization given that the insulin receptor signaling may be more sensitized in the absence of protein aggregation.

### Concluding remarks

Although clinical trials of immunotherapy for AD have been initially disappointing, recent promising results for solanezumab in AD provide hope that improvements in efficacy are forthcoming. Since protein aggregation and related pathology are common features in most neurodegenerative diseases, immunotherapy for PD and other age-related neurodegenerative diseases may also be on the horizon. On the other hand, “anti-insulin resistance” can be viewed as an emerging novel paradigm in PD therapy and related disorders. In this regard, the therapeutic effect of a GLP-1 analog has been already demonstrated in a pilot study of PD^4^ and a parallel study is ongoing in AD. Given this, it is natural to consider whether combining current anti-aggregation immunotherapy with “anti-diabetes” therapy can improve the overall treatment efficacy. Of particular note, “anti-insulin resistance” therapies, such as insulin, GLP-1 analogs, DPP-4 inhibitors, and APN, may improve insulin receptor signaling by up-regulating the activity of proteases such as IDE and KLK6. Taken together, the evidence strongly suggests that the mechanisms of immunotherapy and “anti-insulin resistance” treatments may merge in an additive or even synergistic manner, at the level of suppression of protein aggregation. However, as indicated previously, the neuropathology of neurodegenerative disease (i.e. protein aggregation and inflammation) is often dissociated from the cognitive, behavioral and motor features. Yet, exercise has been the one factor effective in ameliorating cognitive and behavioral abnormalities in both animals and humans and improving pathology. As such, exercise and/or an exercise mimetic, such as APN, may be another particularly important adjunct for immunotherapy as a disease-modifying therapy, and further studies are needed to validate this exciting possibility.
